# *MicroRNA-204* promotes vascular endoplasmic reticulum stress and endothelial dysfunction by targeting Sirtuin1

**DOI:** 10.1038/s41598-017-06721-y

**Published:** 2017-08-24

**Authors:** Modar Kassan, Ajit Vikram, Qiuxia Li, Young-Rae Kim, Santosh Kumar, Mohanad Gabani, Jing Liu, Julia S. Jacobs, Kaikobad Irani

**Affiliations:** Cardiovascular Division, Department of Medicine, and Abboud Cardiovascular Research Center, University of Iowa Carver College of Medicine, IA City, IA 52242 USA

## Abstract

Endoplasmic reticulum (ER) stress has been implicated in vascular endothelial dysfunction of obesity, diabetes, and hypertension. MicroRNAs play an important role in regulating ER stress. Here we show that microRNA-204 (miR-204) promotes vascular ER stress and endothelial dysfunction by targeting the Sirtuin1 (Sirt1) lysine deacetylase. Pharmacologic ER stress induced by tunicamycin upregulates miR-204 and downregulates Sirt1 in the vascular wall/endothelium *in vivo* and in endothelial cells *in vitro*. Inhibition of miR-204 protects against tunicamycin-induced vascular/endothelial ER stress, associated impairment of endothelium-dependent vasorelaxation, and preserves endothelial Sirt1. A miR-204 mimic leads to ER stress and downregulates Sirt1 in endothelial cells. Knockdown of Sirt1 in endothelial cells, and conditional deletion of endothelial Sirt1 in mice, promotes ER stress via upregulation of miR-204, whereas overexpression of Sirt1 in endothelial cells suppresses miR-204-induced ER stress. Furthermore, increase in vascular reactive oxygen species induced by ER stress is mitigated by by miR-204 inhibition. Finally, nutritional stress in the form of a Western diet promotes vascular ER stress through miR-204. These findings show that miR-204 is obligatory for vascular ER stress and ER stress-induced vascular endothelial dysfunction, and that miR-204 promotes vascular ER stress via downregulation of Sirt1.

## Introduction

Endoplasmic reticulum (ER) stress, an adaptive, also termed the unfolded protein response (UPR), is an evolutionarily conserved response. It consists of three main signaling pathways: 1) inositol-requiring protein (IRE1) activation, 2) protein kinase RNA (PKR)-like ER kinase (PERK) activation, and 3) the activation of transcription factor-6 (ATF6)^1^. When adaptation to restore homeostasis fails, ER stress–initiated pathways lead to cellular dysfunction and death^2^. Recent evidence shows that activation of the ER stress response by tradiational risk factos contributes to the pathogenesis of cardiovascular diseases^3, 4^. These studies show that patients and animals with diabetes or hypertension have micro- and macro-vascular complications associated with ER stress induction^5–8^. Although these findings show that alleviating ER stress had beneficial consequences in the vasculature, the mechanism by which ER stress mediates vascular dysfunction are not completely understood.

MicroRNAs (miRs) regulate the ER stress response, either through specific targets or through mechanisms that are yet unclear^9^. MiR-204 was recently implicated in ER stress responsive gene modulation and apoptosis in tumorigenesis^10^. Additionally, miR-211, which shares an almost identical sequence with miR-204, is induced in a PERK-dependent manner^11^. Despite these findings, whether miR-204 leads to vascular dysfunction in an ER stress-dependent manner is not known.

MiR-204 is expressed in vascular cells^12–15^. One of the many targets of miR-204 is the Sirtuin1 lysine deacetylase (Sirt1)^15, 16^. Sirt1 plays vital roles in angiogenensis^17^, endothelium-dependent vasorelaxation^18^, and protects against experimental atherosclerosis^19^. Sirt1 also mitigates endothelial oxidative stress and apoptosis^20, 21^. Furthermore, Sirt1 exerts at least some of its anti-atherosclerotic effect by inhibiting VSMC hypertrophy^22, 23^. However, the relationship between miR-204 and Sirt1 in the context of endothelial dysfunction triggered by ER stress is not known. In this report we investigated if miR-204 is obligatory for endothelial ER stress and leads to vascular endothelial dysfunction by targeting Sirt1.

## Results

### Endothelial miR-204 is induced by and promotes ER stress

**Figure 1:**
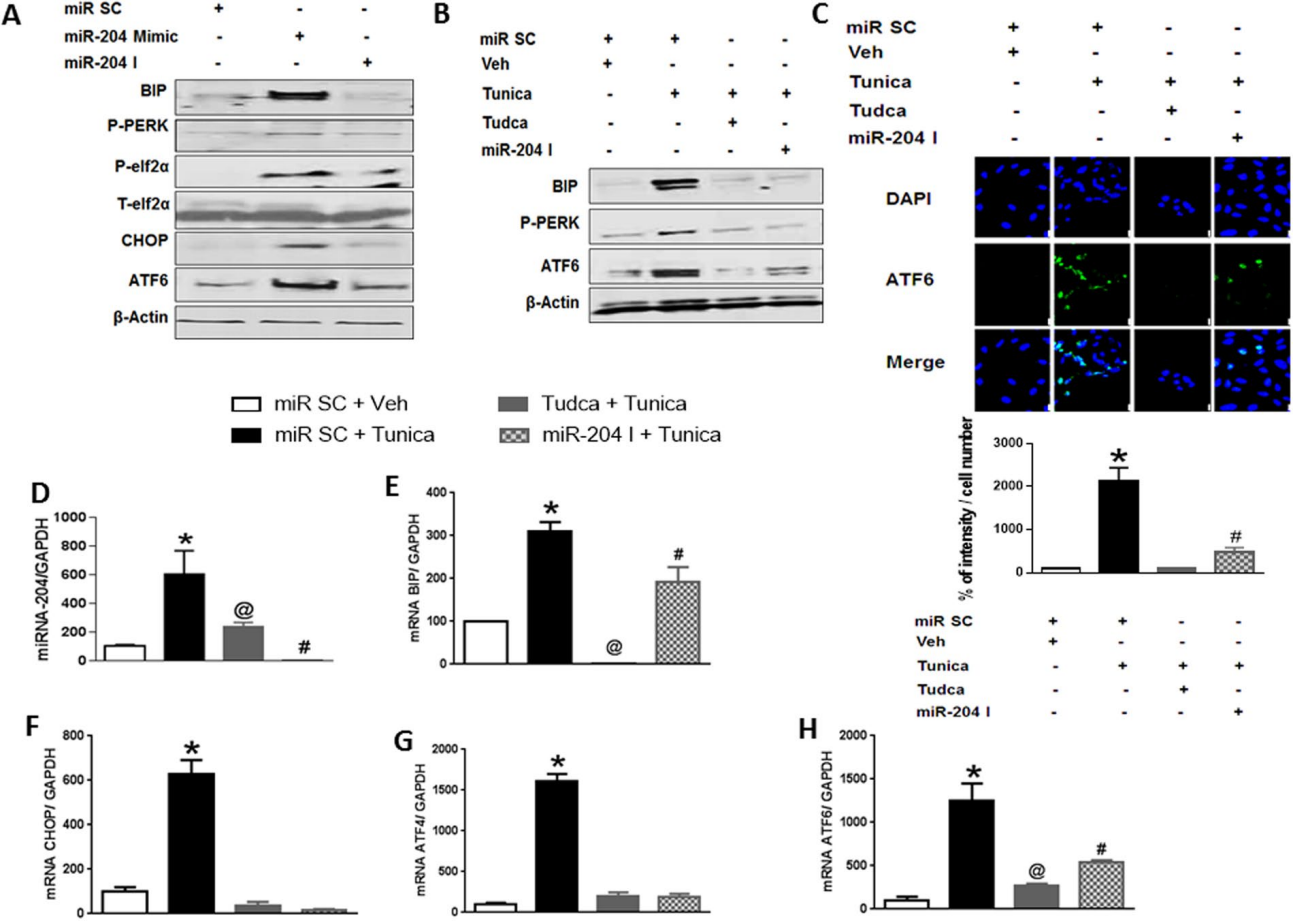
MiR-204 promotes ER stress in endothelial cells. **(A)** miR-204 mimic induces ER stress in endothelial cells. Immunoblots for ER stress markers (BIP, P-PERK, P-elf2α, CHOP, and ATF6) in HUVECs transfected with miR-204 mimic (50 nM for 48 h). Control cells were transfected with a scrambled oligonucleotide (miR SC), or miR-204 inhibitor (miR-204 I). Data is representative of three independent experiments. (**B,C**) Inhibition of miR-204 suppresses tunicamycin-induced expression of ER stress markers in endothelial cells. Immunoblots (**B**) and immunofluorescence with quantification (**C**) for ER stress markers in HUVECs treated with tunicamycin (Tunica, 1 μg/ml for 6 h) and transfected with miR-204 I. Tudca (500 μg/ml for 6 h) was used as an ER stress inhibitor. Data is representative of three independent experiments. Control cells were transfected with miR SC and treated with vehicle (Veh). Values expressed relative to miR SC + Veh. *p < 0.05 vs miR SC + Veh, Tudca + Tunica, and miR-204 I + Tunica. #p < 0.05 vs miR SC + Veh. (**D**) miR-204 is induced by ER stress in endothelial cells. MiR-204 expression quantified by qPCR in HUVECs treated with Tunica or Tudca and transfected with either miR SC or miR-204 I. MiR-204 expression is normalized to GAPDH and expressed relative to miR SC + Veh. n = 3, *p < 0.05 vs miR SC + Veh, Tudca + Tunica, and miR-204 I + Tunica. @p < 0.05 vs miR SC + Veh, and miR-204 I + Tunica. #p < 0.05 vs miR SC + Veh. (**E–H**) Inhibition of miR-204 suppresses tunicamycin-induced ER stress. mRNA expression of ER stress markers quantified by qPCR in HUVEC transfected with miR204 I and treated with Tunica. Values are normalized to GAPDH and expressed relative to miR SC + Veh. n = 3. *p < 0.05 vs miR SC + Veh, Tudca + Tunica, and miR-204 I + Tunica. @p < 0.05 vs miR SC + Veh, and miR-204 I + Tunica. #p < 0.05 vs miR SC + Veh.

To explore the role of miR-204 in endothelial ER stress, we first asked if overexpression of miR-204 induces ER stress in HUVECs. Transfection of HUVECs with a miR-204 mimic, which led to marked increase in miR-204 expression (Supplementary Fig. 1A), resulted in upregulation of the ER stress markers BIP, CHOP, and ATF6, as well as phosphorylation of PERK and elF2α (Fig. 1A; Supplementary Fig. 1B). Next, to determine if endogenous miR-204 mediates ER stress, we inhibited miR-204 in HUVECs with a miR-204 oligonucleotide inhibitor (miR-204 I) and triggered ER stress with the protein glycosylation inhibitor tunicamycin. Tunicamycin-induced ER stress in endothelial cells was mitigated by miR-204 I (Fig. 1B,C,E–H; Supplementary Fig. 1C). In addition, miR-204 was upregulated in HUVECs treated with tunicamycin (Fig. 1D). Tunicamycin was not the only ER stress trigger that acted via miR-204. Endothelial ER stress induced by thapsigargin, a non-competitive inhibitor of the sarco/endoplasmic reticulum Ca^2+^ ATPase, was also suppressed by miR-204 I (Supplementary Fig. 2A–E). Thus, miR-204 is upregulated by external triggers of ER stress, and is required for the ER stress response in endothelial cells *in vitro*.

### MiR-204 mediates ER stress–induced vascular endothelial dysfunction

**Figure 2:**
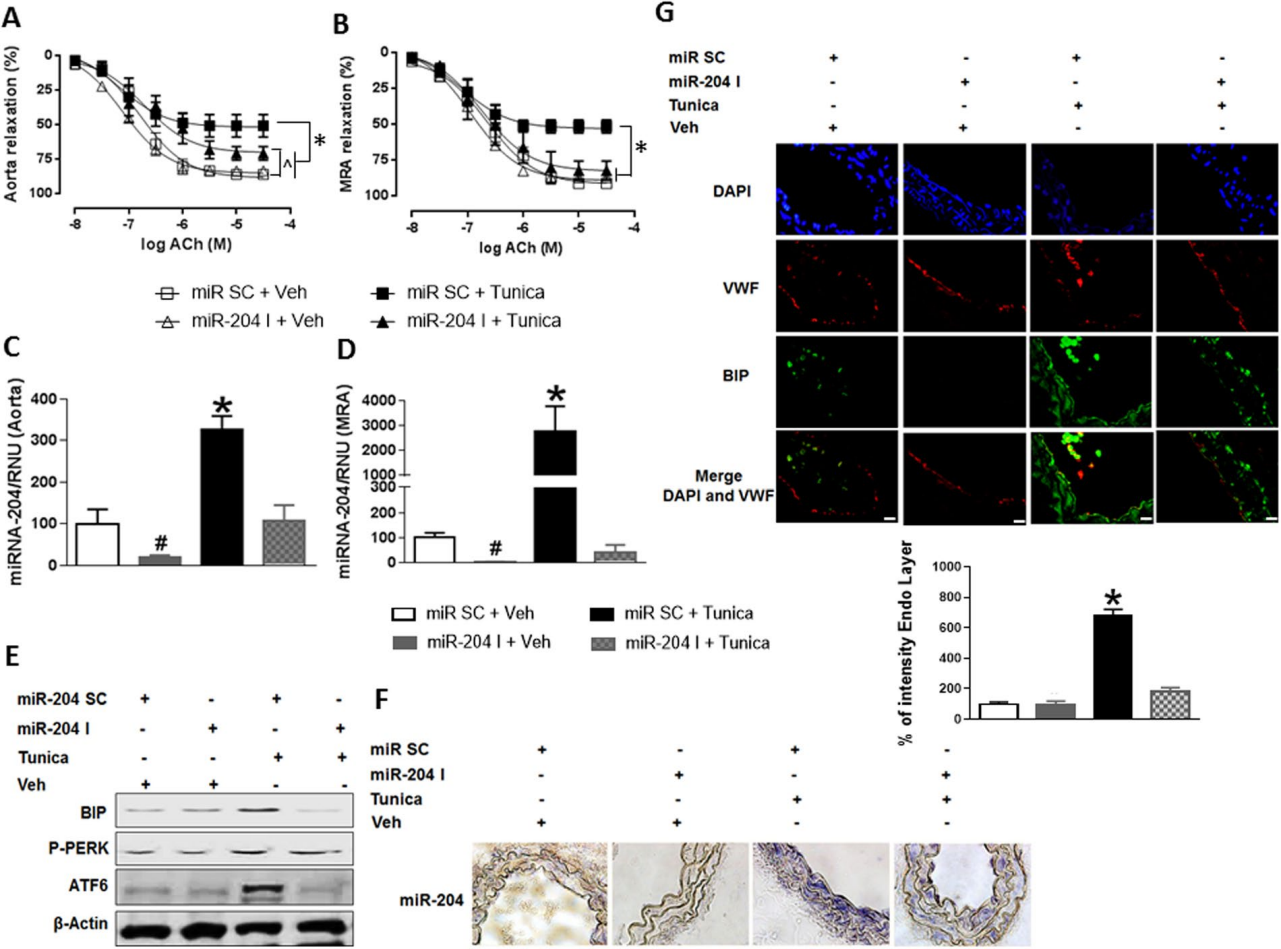
Vascular miR-204 is upregulated by ER stress and promotes endothelial dysfunction. (**A,B**) Systemic infusion with miR-204 inhibitor reverses ER stress-induced endothelial dysfunction. Endothelium-dependent relaxation thoracic aortas **(A)** and mesenteric resistance arteries (MRA) **(B)** of C57/Bl6 mice infused with miR-204 I (0.2 mg/mouse/week) and treated with tunicamycin (0.75 mg/kg, 2 injections/week for two weeks). Control vessels were harvested from mice treated with Veh and infused with miR SC. n = 5. *p < 0.05 vs miR SC + Veh, miR-204 I + Veh, and miR-204 I + Tunica. ^p < 0.05 miR-204 I + Tunica vs miR SC + Veh. (**C–G**) Systemic infusion with miR-204 I reverses Tunica-induced vascular ER stress. MiR-204 expression quantified by qPCR in thoracic aortas (**C**) and MRAs (**D**) of mice infused with miR-204 I and treated with Tunica. Values are normalized to RNU6 and expressed relative to miR SC + Veh. Immunoblots for ER stress markers (**E**), *in situ* hybridization for miR-204 (**F**), and immunofluorescence and quantification for BIP (**G**) in MRAs of mice from **A–D** above. Data is representative of three independent experiments. Von Willebrand Factor (VWF) was used as endothelial marker. BIP expression shown relative to miR SC + Veh. *p < 0.05 vs miR SC + Veh, miR-204 I + Veh, and miR-204 I + Tunica. #p < 0.05 vs miR SC + Veh.

We next determined if miR-204 is responsible for vascular endothelial dysfunction secondary to ER stress. We chose impairment of endothelium-dependent vasorelaxation as a marker of endothelial dysfunction. Mice were systemically infused with miR-204 I or a scrambled oligonucleotide and injected with tunicamycin. Treatment with tunicamycin led to impairment of endothelium-dependent relaxation (Fig. 2A,B), upregulation of miR-204 (Fig. 2C,D,F), and ER stress (Fig. 2E,G; Supplementary Fig. 3E) in aortas and mesenteric resistance arteries (MRAs). Systemic infusion of miR-204 I protected mice from tunicamycin-induced endothelial dysfunction (Fig. 2A,B), suppressed tunicamycin-induced vascular miR-204 (Fig. 2C,D,F), and mitigated tunicamycin-induced vascular ER stress (Fig. 2E,G; Supplementary Fig. 3E). The contractile response to phenylephrine (Supplementary Fig. 3A,C) and endothelial-independent relaxation to sodium nitroprusside (Supplementary Fig. 3B,D) were similar among groups. Therefore, vascular miR-204 is upregulated with ER stress *in vivo*, and mediates ER stress-induced endothelial dysfunction.

### Downregulation of Sirt1 promotes endothelial ER stress

**Figure 3:**
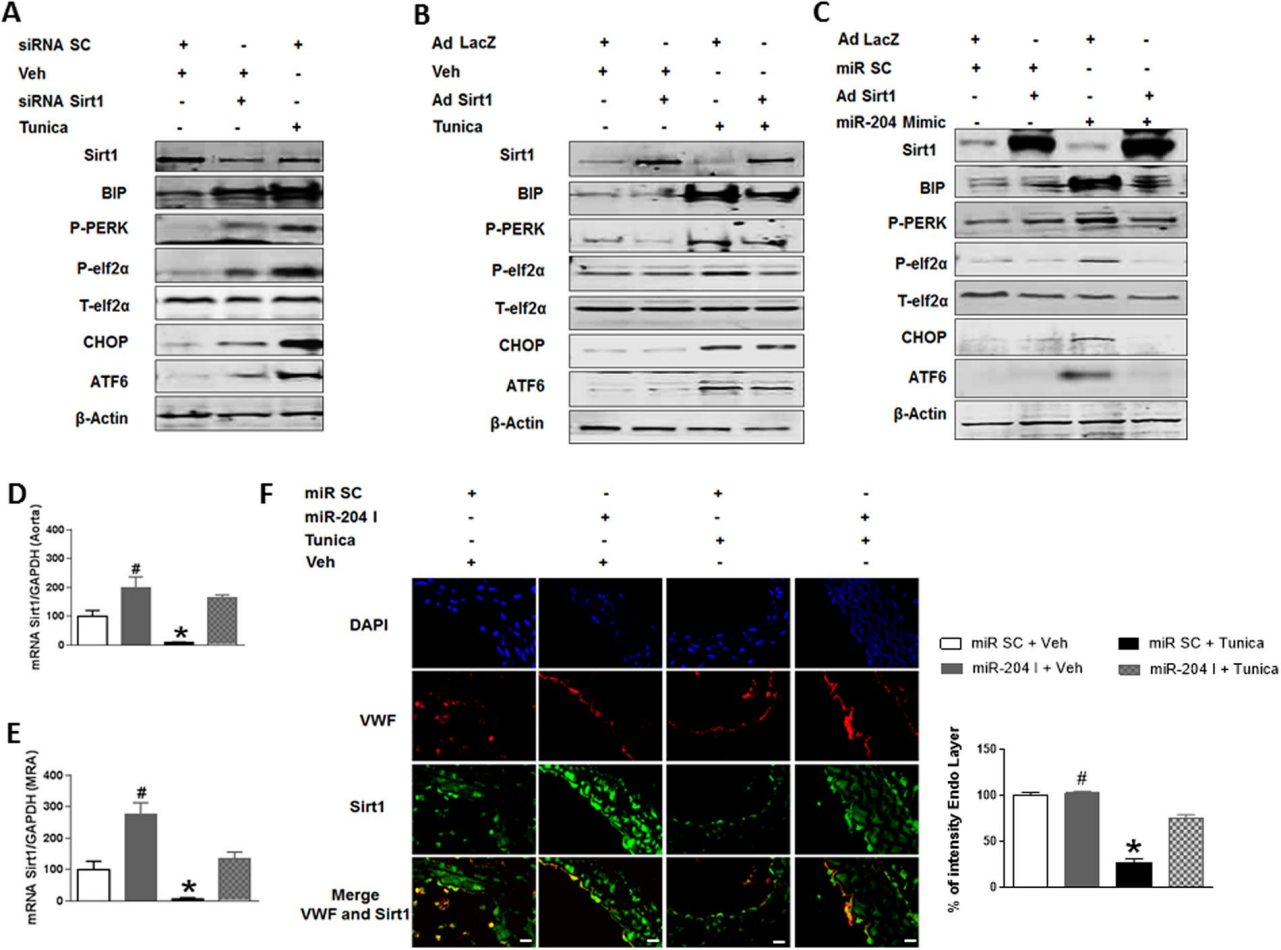
Sirt1 downregulation by miR-204 promotes ER stress. (**A**) Immunoblots for ER stress markers in HUVECs transfected with siRNA Sirt1 (20 pmol) or treated with Tunica. Control cells were transfected with a scrambled siRNA (siRNA SC) or treated with vehicle (Veh). Data is representative of three independent experiments. (**B**) Immunoblots for ER stress markers and Sirt1 in HUVECs transfected with Sirt1 adenovirus (Ad Sirt1) and treated with Tunica. Control cells were infected with an adenovirus expressing LacZ (Ad LacZ) and treated with Veh. Data is representative of three independent experiments. (**C**) Immunoblots for ER stress markers in HUVECs transfected with miR-204 mimic (50 nM for 48 h) and infected with Ad Sirt1. Data is representative of three independent experiments. Sirt1 quantified by qPCR in thoracic aorta (**D**) and by qPCR and immunofluorescence in MRAs (**E,F**) of mice systemically infused with miR-204 I (0.2 mg/mouse/week) and treated with Tunica. Sirt1 mRNA is normalized to GAPDH and expressed relative to miR SC + Veh. *p < 0.05 vs miR SC + Veh, miR-204 I + Tunica, and miR-204 I + Veh. #p < 0.05 vs miR SC + Tunica. vWF: Von Willebrand Factor.

We then inquired into the relationship between miR-204 and Sirt1 in the context of ER stress. We first investigated if Sirt1, independent of miR-204, regulates endothelial ER stress pathways. Short interfering RNA-mediated knockdown Sirt1 in HUVECs promoted ER stress (Fig. 3A; Supplementary Fig. 4A), and exacerbated tunicamycin-induced ER stress (Supplementary Fig. 4E), as evidenced by upregulation of ER stress markers. In addition, treatment with tunicamycin resulted in downregulation of Sirt1 and upregulation of miR-204 in HUVECs (Fig. 3A,B; Supplementary Fig. 4A,B,D,F and H), and in mouse aortas and MRAs (Fig. 3D–F). Because miR-204 is in intron 6 of the *TRPM3* gene, and is co-transcribed with *TRPM3*, we also measured TRPM3 expression. Paralleling miR-204 expression, TRPM3 was also upregulated in HUVECs treated with tunicamycin (Supplementary Fig. 4I). Thus, endogenous Sirt1 plays an important role in suppressing endothelial miR-204 and ER stress.

### Overexpression of Sirt1 inhibits endothelial ER stress

We next asked if Sirt1 overexpression suppresses endothelial ER stress. Overexpression of Sirt1 (lacking the 3′-UTR) in HUVECs using a recombinant adenovirus (Ad Sirt1) inhibited tunicamycin-induced ER stress (Fig. 3B; Supplementary Fig. 4B,F and G), and suppressed tunicamycin-induced miR-204 and TRPM3 expression (Supplementary Fig. 4H and I). To further investigate whether the protection from ER stress conferred by Sirt1 is specific to tunicamycin, we examined the effect of Sirt1 on thapsigargin-induced ER stress. Sirt1 overexpression in HUVECs suppressed ER stress induced by thapsigargin (Supplementary Fig. 5A–E). Furthermore, overexpression of Sirt1 inhibits endothelial ER stress induced by transfection of miR-204 mimic (Fig. 3C; Supplementary Fig. 4C,J and K). Importantly, overexpression of Sirt1 did not alter total (exogenous mimic plus endogenous) miR-204 (Supplementary Fig. 4L). These findings show that ER stress downregulates endothelial Sirt1, and restoring Sirt1 suppresses ER stress triggered by external stimuli as well as by overexpression of miR-204.

### Conditional deletion of endothelial Sirt1 in mice promotes ER stress and endothelial dysfunction through miR-204

**Figure 4:**
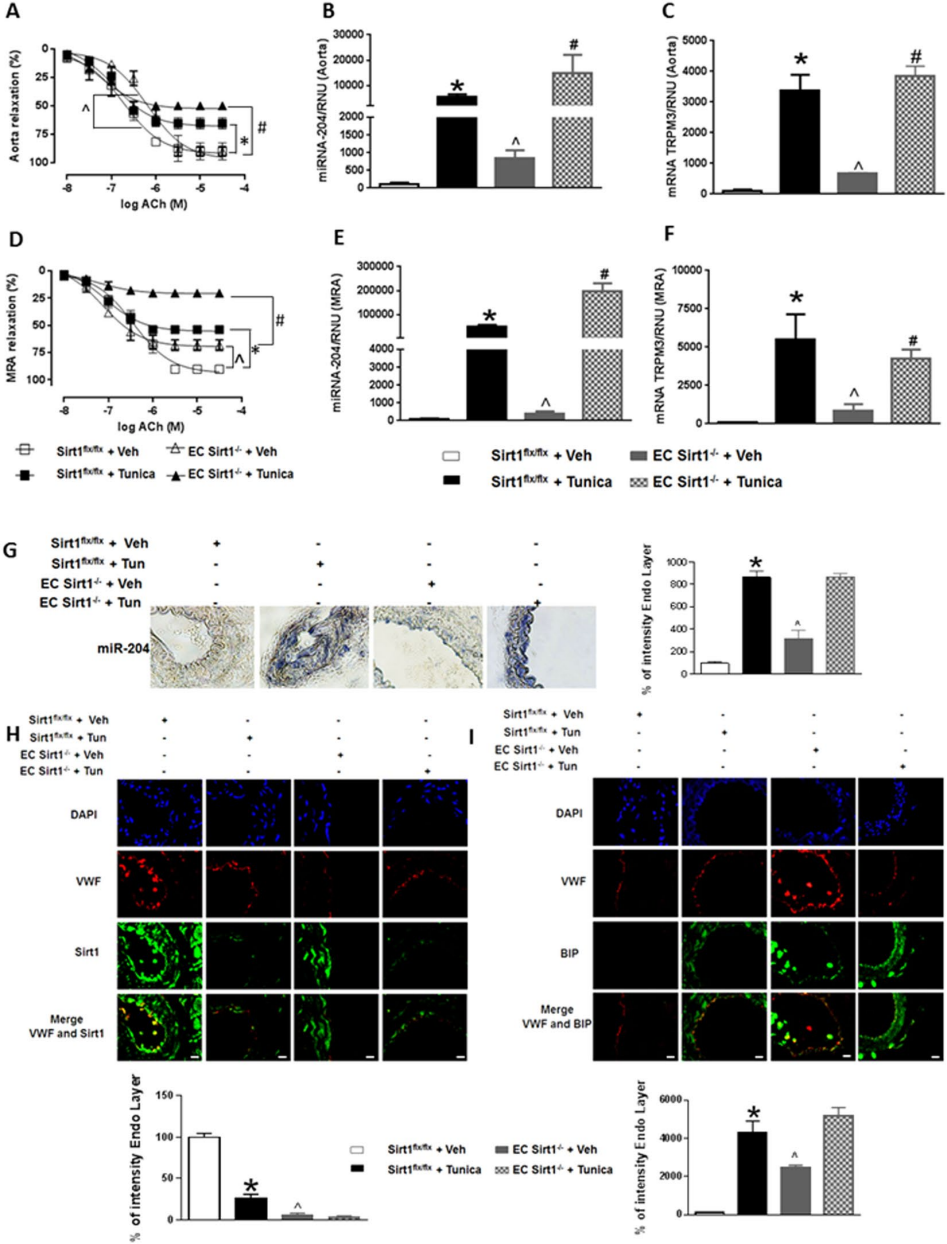
Conditional deletion of endothelial Sirt1 upregulates miR-204, promotes vascular ER stress and leads to endothelial dysfunction. Endothelial-dependent relaxation to acetylcholine (Ach) in thoracic aortas (**A**) and MRAs (**D**) of EC Sirt1^−/−^ and control Sirt1^flx/flx^ mice with and without Tunica treatment. n = 5. qPCR for miR-204 and TRPM3 in thoracic aortas (**B,C**) and MRAs (**E,F**) of EC Sirt1^−/−^ and Sirt1^flx/flx^ mice with and without treatment with Tunica. Values are normalized to RNU and expressed relative to Sirt1^flx/flx^ + Veh. n = 3. *p < 0.05 vs Sirt1^flx/flx^ + Tunica. #p < 0.05 vs EC Sirt1^−/−^ + Tunica. ^p < 0.05 vs EC Sirt1^−/−^ + Veh. *In situ* hybridization and quantification for miR-204 (**G**) and immunofluorescence with quantification for Sirt1 (**H**) and BIP (**I**) in MRAs of EC Sirt1^−/−^ and Sirt1^flx/flx^ mice with and without treatment with Tunica. Von Willebrand Factor (VWF) was used as endothelial marker. n = 3. Values expressed relative to Sirt1^flx/flx^ + Veh. *p < 0.05 vs Sirt1^flx/flx^ + Tunica. ^p < 0.05 vs EC Sirt1^−/−^ + Veh.

To further understand the relationship between endothelial Sirt1 and miR-204 as it applies to vascular ER stress, we generated mice with conditional deletion of endothelial Sirt1 by crossing Sirt1^flx/flx^ mice with cadherin-5-Cre mice (EC Sirt1^−/−^; Fig. 4H). Compared with Sirt1^flx/flx^ mice, these mice have impaired endothelium-dependent vasorelaxation in both their aortas and MRAs, and this impairment was exacerbated by treatment with tunicamycin (Fig. 4A,D). In addition, EC Sirt1^−/−^ mice have elevated miR-204 and TRPM3 in aortas and MRAs compared with Sirt1^flx/flx^ mice (Fig. 4B,C,E, and F). *In situ* hybridization and immunohistochemistry showed upregulation of miR-204 and ER stress markers throughout the thickness of the vessel wall, including the endothelium (Fig. 4G,I). Moreover, in addition to basal upregulation of miR-204 and ER stress, arteries of EC Sirt1^−/−^ showed exaggerated induction of miR-204 and ER stress markers (Fig. 4G,I), and downregulation of vascular Sirt1 (Fig. 4H), in response to tunicamycin treatment.

**Figure 5:**
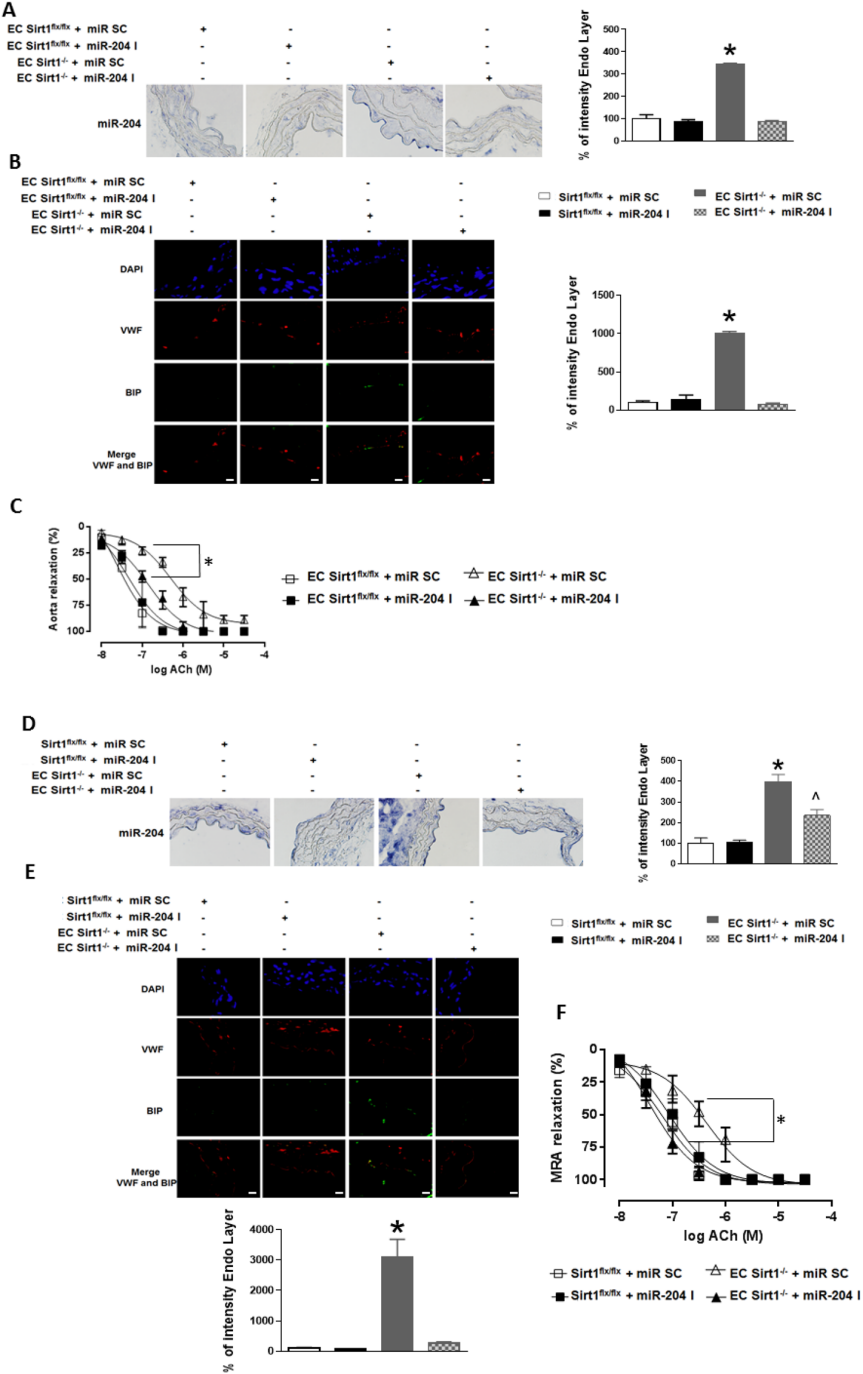
Vascular ER stress and endothelial dysfunction in mice due to conditional deletion of endothelial Sirt1 is mediated by miR-204. *In situ* hybridization with quantification for miR-204 (**A,D**), immunofluorescence with quantification for BIP (**B,E**), and endothelium-dependent relaxation in thoracic aortas (**A–C**) and MRAs (**D–F**) of EC Sirt1^−/−^ and Sirt1^flx/flx^ mice transfected *ex vivo* with miR-204 I or miR SC. n = 3. Values expressed relative to Sirt1^flx/flx^ + miR SC. *p < 0.05 vs Sirt1^flx/flx^ + miR SC, Sirt1^flx/flx^ + miR-204 I, and EC Sirt1^−/−^ + miR-204 I.

We then investigated if miR-204 is obligatory for vascular ER stress and endothelial dysfunction in EC Sirt1^−/−^ mice. We performed *ex vivo* transfections of thoracic aortas and MRAs isolated from EC Sirt1^−/−^ mice with miR-204 I, which resulted in downregulation of miR-204 (Fig. 5A,D), suppression of ER stress (Fig. 5B,E), and rescue of endothelium-dependent vasorelaxation (Fig. 5C,F). Collectively, these findings illustrate that endothelial Sirt1 protects the vasculature from ER stress and maintains vascular homeostasis by curtailing miR-204 expression.

### MiR-204 is required for ER stress-induced reactive oxygen species

**Figure 6:**
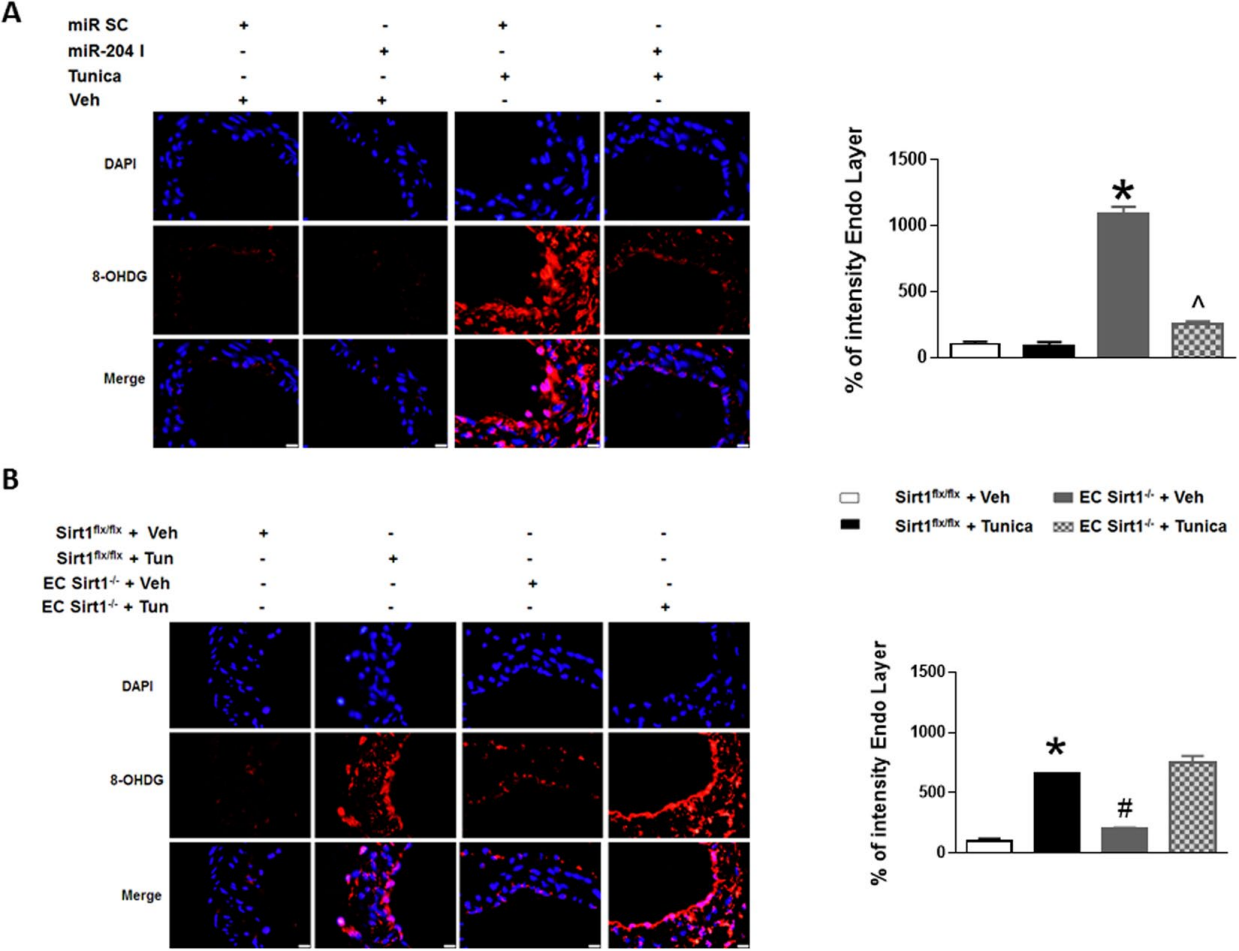
MiR-204 promotes and Sirt1 suppresses ER stress-induced vascular reactive oxygen species. Immunofluorescence and quantification for endothelial 8-hydroxydeoxyguanosine (8-OHdG) in MRAs of C57Bl/6 mice systemically infused with miR-204 I and treated with Tunica or Veh (**A**), and EC Sirt1^−/−^ or Sirt1^flx/flx^ mice treated with Tunica or Veh (**B**). Values expressed relative to Sirt1^flx/flx^ + Veh. miR SC + Veh: white bar, miR SC + miR-204 I: black bar, miR SC + Tunica: grey bar, miR-204 I + Tunica: checkered bar. *p < 0.05 vs miR SC + Veh, and miR-204 I + Veh (in **A**), and Sirt1^flx/flx^ + Veh (in **B**). ^p<0.05 vs miR SC + Tunica. #p<0.05 vs EC Sirt1^−/−^ + Veh. n = 3.

Change in the redox state with ER stress is well-documented^24, 25^. ER stress increases reactive oxygen species (ROS) leading to oxidative stress^26, 27^. We investigated if miR-204 mediates ER stress-induced ROS. We first determined the effect of miR-204 antagonism on tunicamycin-stimulated 8-hydroxydeoxyguanosine (8-OHDG), an oxidative adduct of DNA. Compared with mice infused with scrambled oligonucleotide, infusion of miR-204 I decreased 8-OHDG in MRAs of tunicamycin-treated mice (Fig. 6A). In addition, both basal and tunicamycin-stimulated 8-OHDG was upregulated in MRAs of EC Sirt1^−/−^ mice comparted with EC Sirt1^flx/flx^ mice (Fig. 6B). These findings identify an important role for miR-204 in promoting, and endothelial Sirt1 in suppressing, ER stress-induced vascular/endothelial ROS.

### MiR-204 regulates western diet-stimulated vascular ER stress and endothelial dysfunction

**Figure 7:**
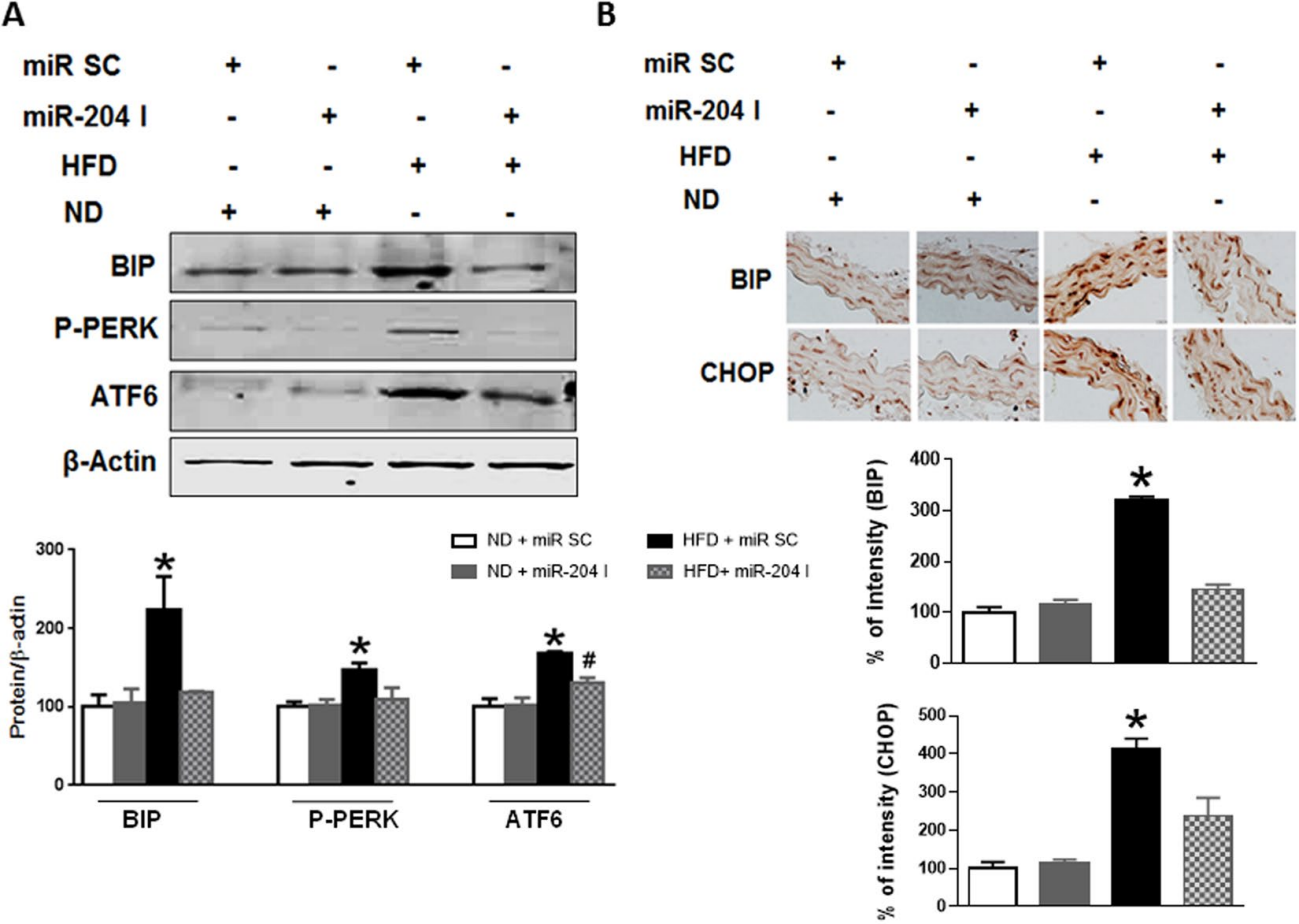
High-fat diet promotes vascular ER stress through miR-204. Immunoblots with quantification for BIP, p-PERK and ATF6 (**A**) and immunohistochemistry with quantification for BIP and CHOP (**B**) in aortas of C57Bl/6 mice fed a high-fat diet (HFD) and infused with miR-204 I or miR SC. Data is representative of three independent experiments and expressed relative to ND + miR SC. n = 3. *p < 0.05 vs ND + siRNA SC, ND + miR-204 I, and HFD + miR-204 I. #p < 0.05 vs ND + miR SC.

To put our findings into a more physiological context, we inquired into the relationship between vascular miR-204 and ER stress in the setting of diet-induced obesity, a condition associated with vascular endothelial dysfunction. ER stress was induced in aortas of mice fed a western high-fat diet (Fig. 7). This increase was significantly lower in mice systemically infused with miR-204 I compared with those infused with scrambled oligonucleotide (Fig. 7). Thus, miR-204 is responsible for vascular ER stress in diet-induced obesity, a condition that is associated with vascular endothelial dysfunction.

## Discussion

Our findings add to the growing appreciation of the role of miR-204 in ER stress in general, and vascular ER stress in particular. Prior studies have shown that miR-204 promotes vascular ER stress by downregulating endothelial Caveolin-1, and Sirt1 protects Caveolin-1 from miR-204-mediated downregulation^28, 29^. The present study goes further by demonstrating that miR-204-mediated vascular ER stress is also due to downregulation of Sirt1. It also corroborates prior work, demonstrating crosstalk between miR-204 and Sirt1, by showing that miR-204 promotes ER stress by targeting Sirt1, while Sirt1 suppresses ER stress by downregulating miR-204. While Sirt1 may suppress endothelial ER stress through molecular mechanisms described in other tissues and cell types, such as in the liver and hepatoma cells^30, 31^, Sirt1-dependent downregulation of miR-204 may represent an additional mechanism by which it inhibits ER stress.

How precisely Sirt1 controls expression of miR-204, and whether this control involves transcriptional or epigenetic regulation of miR-204 (transcribed from the *TRPM3* locus) or through indirect mechanisms, is not clear. However, it is worth noting that thapsigargin-induced upregulation of miR-211, a closely related microRNA which differs from miR-204 by only one nucleotide but shares the same seed sequence (UUUCCCU), in mouse embryonic fibroblasts is dependent on PERK^32^. Moreover, Sirt1 inhibits the PERK/elF2α arm of the ER stress response^33^. These findings, taken together, raise the possibility that Sirt1-dependent inhibition of PERK may contribute to mitigation of miR-204 induction by ER stress in endothelial cells. Regardless of the mechanism, these findings paint a feed forward relationship in which downregulation of Sirt1 by miR-204 during ER stress contributes to amplification of miR-204, and miR-204 promotes ER stress by targeting Sirt1 (Supplementary Fig. 6).

A prior studiy showed that miR-204 promotes ER stress in pancreatic beta-cells^11^. Furthermore, both miR-204 and miR-211, which is encoded in the *TRPM1* gene and transcribed independently from miR-204, are upregulated with thapsigargin and low glucose-stimulated ER stress in mouse embryonic fibroblasts^34^. Although the functional significance and targets of miR-204 upregulation in fibroblasts was not explored, miR-211 downregulated the ER stress protein CHOP by an unusual mechanism involving transcriptional repression of the CHOP promoter, thereby functioning as a pro-survival microRNA during ER stress. While we did not measure miR-211, or examine its role in endothelial ER stress, the above-mentioned mechanism could be operative in endothelial cells as well, and may counteract the role of miR-204 in inducing terminal ER stress.

In summary, this work identifies a novel interactome involving Sirt1 and miR-204 in the regulation of vascular ER stress and vascular endothelial function. Further, this work provides evidence that miR-204 serves as a conduit through which environmental stressors such as a high-fat diet that lead to ER stress contribute to vascular endothelial dysfunction. While this work was limited to endothelial cells and the vasculature, and was focused exclusively on ER stress, the relationship between miR-204 and Sirt1 described herein may also be relevant in other cell types and tissues that express Sirt1 and miR-204, and may play a part in other fundmental cellular processes such as autophagy which are regulated by both Sirt1 and miR-204^29, 35^.

## Methods

### Animals

Experiments were performed on 8–12 week-old 1) C57Bl/6 mice and 2) mice with conditional deletion of endothelial Sirt1 (EC Sirt1^−/−^). EC Sirt1^−/−^ mice were generated by crossing Sirt1^flx/flx^ mice with cadherin-5-Cre mice in which the Cre recombinase is driven by an endothelium-specific cadherin-5 promoter. Sirt1^flx/flx^ mice were used as controls. Mice were fed standard chow (Research Diets Inc., New Brunswick, NJ) containing (in kilocalories) 10% fat, 70% carbohydrate, and 20% protein (D12450B). Terminal experiments were performed in anesthetized mice (2–5% isoflurane). The thoracic aorta and the mesenteric resistance arteries were isolated and used for immunoblotting, immunostaining, *in situ* hybridization (ISH), real-time quantitative polymerase chain reaction (qPCR), and vascular reactivity. All protocols were approved by the Institutional Animal Care and Use Committee of the University of Iowa. All methods were performed in accordance with the guidelines and regulations of the NIH and University of Iowa.

### *In vivo* induction of ER stress by tunicamycin

Tunicamycin (Calbiochem, San Diego, CA USA) was dissolve in dimethyl sulfoxide (DMSO) and was injected intraperitoneally at a dose of 0.75 mg/kg, two injections per week for 2 weeks, into mice as previously described^36^. DMSO vehicle was used in control mice.

### HFD (Western diet)-induced model of endothelial dysfunction

Mice were fed a HFD (Envigo, TD.88137) for 8 weeks. Mice fed a normal diet (ND) were used as controls.

### *In vivo* inhibition of miR-204

Locked nucleic acid miR-204 inhibitor (5′-AGG ATG ACA AAG GGA-3′) or a scrambled nucleotide (5′-ACG TCT ATA CGC CCA-3′) (Ambion Life Technologies, Grand Island, NY, USA) was infused into mice using ALZET osmotic mini-pumps that were implanted subcutaneously in mice. Mice treated with tunicamycin were infused with miR-204 inhibitor or scrambled nucleotide for 2 weeks (0.2 mg/week/mouse). HFD and ND fed mice were infused with miR-204 inhibitor or scrambled nucleotide for 6 weeks (0.7 mg/kg/day).

### *Ex vivo* infections with adenoviruses and transfections with miR-204 inhibitor

MiR-204 inhibitor, scrambled nucleotide, and recombinant adenoviruses encoding the *E. Coli LacZ* gene (Ad LacZ) or Sirt1 (Ad Sirt1) were transfected into freshly isolated thoracic aortas and mesenteric resistance arteries from mice. Oligonucleotides were transfected after incubation with oligofectamine. Studies were performed 24 h after transfection.

### Mouse vascular reactivity

Male mice 8–12 weeks old were anesthetized and euthanized by rapid cardiac excision. The thoracic aorta and the MRA were carefully harvested and placed in ice-cold Krebs buffer (118.3 mM NaCl, 4.7 mM KCl, 2.5 mM CaCl_2_, 1.2 mM KH_2_PO_4_, 25 mM NaHCO_3_, 1.2 mM MgSO_4_, 11 mM glucose, 0.0026 mM CaNa_2_ EDTA). The vessels were cleaned of fat and connective tissue and cut transversely into 5–10 rings (1.8–2.0 mm wide). The rings were placed in oxygenated chambers (95% O_2_/5% CO_2_) filled with 5 mL Krebs buffer solution and maintained at 37 °C and pH 7.4. Each ring was suspended between two wire stirrups in a 5 mL chamber of a four-chamber myograph system (DMT). One stirrup was connected to a three–dimensional micromanipulator and the other to a force transducer. The contractile force was recorded electronically. All rings were stretched to 2000 mg in 500 mg increments over a 1 h period to optimize the contractile response to KCl. One dose of KCl (60 mM) was added to verify vascular smooth muscle viability. Cumulative dose–response curve to phenylephrine (PE) (10^−9^ to 10^−5^ M) was obtained by administering the drug in one-half log doses. Endothelium-dependent vasodilatation was determined by generating dose–response curves to acetylcholine (ACh) (10^−9^ to 10^−5^ M). Vasorelaxation evoked by ACh was expressed as percent of maximal contraction. Endothelium-independent vasodilatation was measured by vasorelaxation evoked by cumulative doses of sodium nitroprusside (SNP) in rings pre-constricted with phenylephrine (10^−6^ M) and was also expressed as percent maximal contraction.

### Immunostaining

Paraffin blocks of aortas and MRAs were de-paraffinized with xylene followed by antigen retrieval by heating in citrate buffer (10 mM). Sections were probed with appropriate primary antibodies. Sirt1 antibody (Santa Cruz Biotech, Dallas, TX), Binding immunoglobulin protein (BIP), and C/EBP homologous protein (CHOP) antibodies (Cell Signaling, Denver, MA, USA), von Willebrand factor antibody and 8-hydroxydeoxyguanosine (8-OHdG) antibodies (Abcam, Cambridge, MA,) were used at a 1:50 to 1:200 dilution followed by a biotinylated secondary antibody for immunofluorescence, or streptavidin peroxidase solution, DAB peroxidase substrate, and hematoxylin counterstain for immunohistochemistry. Sections were digitally imaged with an Olympus BX-61 microscope. Images of vessels and cells were taken at 40 × and 60 × magnification, respectively.

### Western blotting

Immunoblotting was performed on aortic and MRA lysates as previously described^37^. Chemiluminescent signal was developed using the Licor Odyssey Scanner (Lincoln, NE, USA). Bands were quantified using image J.

### *In situ* hybridization (ISH) for miR-204

Aortic and MRA sections were de-paraffinized with xylene, followed by Proteinase K treatment (10 μg/mL for 5 min). ISH buffer (Exiqon, Vedbaek, Denmark; production #90000) was added with miR-204 probe (Exiqon, 5′-Dig-N-AGG CAT AGG ATG ACA AAG GGA A-N-Dig-3′) or with a scrambled miR probe (Exiqon, 5′-Dig-N-GTG TAA CAC GTC TAT ACG CCC A-N-Dig-3′) at 20 nM or 40 nM, and incubated for 72 h at 56 °C. After washing, sections were incubated in blocking solution for 15 min (5 mL PBS + 50 mg BSA + 100 uL Sheep serum + 2.5 uL Tween 20), followed by incubation with anti-DIG-FAB overnight (1:800 in antibody dilution solution). Slides were dipped in a solution containing BCIP/NBT (Roche, Mannheim, Germany) and incubated at 30 °C for 48 h. The slides were mounted with DPX and observed under the microscope.

### Cell culture, plasmid/siRNA transfections, and adenoviral infections

Human umbilical vein endothelial cells (HUVECs) were purchased from Clonetics (San Diego, CAUSA) and cultured in endothelial growth medium (EGM-2, Lonza, Walkersville, MD, USA). Cells were treated with tunicamycin (Tunica, 1 μg/mL for 6 h) or Thapsigargin (TPS, 1 μM for 4 h). Cells were transfected with plasmids, miR-204 mimic (5′-UUC CCU UUG UCA UCC UAU GCC U-3′), miR-204 inhibitor (5′-AGG ATG ACA AAG GGA-3′), scrambled oligonucleotide (5′-ACG TCT ATA CGC CCA- 3′), validated Sirt1 siRNA, or negative control siRNA purchased from Invitrogen, with Lipofectamine 2000 (Invitrogen, Carlsbad, CA). Cells were infected with 6 × 10^11^ viral particles/ml of the control Ad LacZ or the Ad Sirt1 adenoviral stocks and incubated at 37 °C for 24 h.

### Quantitative real time PCR

Total RNA was isolated by the acid guanidinium thiocyanate/phenol/chloroform method. Total RNA from cultured cells or tissues of mice was isolated by the TRIZOL (Invitrogen) method. Real-time PCR was performed using the Prism 7000 Sequence Detection System (Applied Biosystems, Foster City, CA) with the SuperScript III Platinum SYBR Green One-Step qRT-PCR Kit (Invitrogen). The following primers purchased from Exiqon were used: Mouse Sirt1 forward 5′-AATGCTGGCCTAATAGACTTGCA-3′, reverse 5′-CCGTGGAATATGTAACGATTTGG-3′; human Sirt1 forward 5′-TCGCAACTATACCCAGAACATAGACA-3′, reverse 5′-CTGTTGCAAAGGAACCATGACA-3′; human GAPDH: forward 5′-ATGACATCAAGAAGGTGGTG-3′; reverse 5′-CATACCAGGAAATGAGCTTG-3′; mouse GAPDH forward 5′-GGCAAATTCAACGGCACA-3′, reverse 5′-CGCTCCTGGAAGATGGTGAT-3′; human ATF6 forward 5′-CAGCGCCCAAGACTCAAA-3′, reverse 5′-TTTGAATGATGATGGCTT TTG-3′; human ATF4 forward 5′-CGATGCTCTGTTTCGAATGGA-3′, reverse 5′-CCAACGTGGTCAAGAGCTCAT-3′; human CHOP forward 5′-AGGAGCCAGGGCCAACA-3′, reverse 5′-TCTGGAGAGCGAGGGCTTT-3′; human GRP78 forward 5-GCTCGACTCGAATTCCAAAG-3′, reverse 5- TTTGTCAGGGGTCTTTCACC-3′. human TRPM3 forward 5′-CGCAGCTGGAAGACCTTATC-3′, reverse 5′-AAGCTGCTCTGACGGACAAT-3′; mouse TRPM3 forward 5′-ACCCCGTCAAGTAGTG-3′, reverse 5′-CCCCAAAGTTGGCGT-3′. MiR-204 forward and universal reverse primers were procured from Quanta Biosciences (cDNA synthesis kit, Quanta Biosciences, Beverly, MA, USA). Mouse GAPDH and RNU6 (Quanta Biosciences, Beverly, MA, USA) were used as internal controls.

### Statistical analysis

Statistical analysis was performed using GraphPad Prism (Version 6.0) statistical software. Significance of difference between two groups was evaluated using the *t*-test. For multiple comparisons, one-way analysis of variance (ANOVA) was used and *post-hoc* analysis was performed with Tukey’s test. Date are expressed as mean ± SEM and considered significant if *P-*values were ≤ 0.05. All shown data is representative of at least three independent experiments.

## Electronic supplementary material

Supplementary Figures and Legends
