# Supplementary material for: MicroRNA-204 promotes vascular endoplasmic reticulum stress and endothelial dysfunction by targeting Sirtuin1
Source: Sci Rep. 2017 Aug 24;7:9308. doi: 10.1038/s41598-017-06721-y (PMC5571183; doi:10.1038/s41598-017-06721-y)
Supplement: Supplementary file 1 — Supplementary Figures and Legends [file 41598_2017_6721_MOESM1_ESM.pdf]

MicroRNA-204 promotes vascular endoplasmic reticulum stress and endothelial dysfunction by targeting Sirtuin1

Modar Kassan[1], Ajit Vikram[1], Qiuxia Li[1], Young-Rae Kim[1], Santosh Kumar[1], Mohanad Gabani[1], Jing Liu[1], Julia S. Jacobs[1], and Kaikobad Irani[1].

1. Cardiovascular Division, Department of Medicine, University of Iowa, and Abboud Cardiovascular Research Center, University of Iowa Carver College of Medicine

Correspondence to: modar-kassan@uiowa.edu or kaikobad-irani@uiowa.edu.

Supplementary Figure Legends

Supplementary Fig. 1. MiR-204 promotes ER stress. (**A**) MiR-204 quantified by qPCR in HUVECs transfected with miR-204 mimic and scrambled control miR (miR SC). MiR-204 is normalized to RNU6 and values expressed relative to time 0. *p<0.05 vs time 0. n = 3. (**B**) Quantification of immunoblots for ER stress markers (BIP, P-PERK, P-eIf2α, CHOP, and ATF6) in HUVECs transfected with miR-204 mimic, miR SC or miR-204 inhibitor (miR-204 I). Values are normalized to β-actin and expressed relative to miR SC. *p<0.05 vs miR SC and miR-204 I. n = 3. (**C**) Quantification of immunoblots for ER stress markers (BIP, P-PERK, and ATF6) in HUVECs transfected with miR-204 I or miR SC and treated with tunicamycin (Tunica) or vehicle control (Veh). Values are normalized to β-actin and expressed relative to miR SC + Veh. *p<0.05 vs miR SC + Veh, Tudca + Tunica, and Tunica + miR-204 I. n = 3.

Supplementary Fig. 2. MiR-204 is responsible for thapsigargin-induced ER stress. (A) Immunoblots for ER stress markers (BIP, P-PERK, and ATF6) in HUVECs transfected with miR-204 I and treated with thapsigargin (TPS). Data is representative of three independent experiments. (**B-E**) qPCR for ER stress markers (BIP, CHOP, ATF4, and ATF6) in HUVECs transfected with miR-204 I and treated with TPS. Values are normalized to GAPDH and expressed relative to miR SC + Veh. *p<0.05 vs miR SC + Veh, miR-204 I + Veh, and miR-204 I + TPS. n = 3.

Supplementary Fig. 3. ER stress does not affect endothelium-independent vascular function. Contraction to cumulative doses of phenylephrine (PE) and endothelial-independent relaxation to cumulative doses of sodium nitroprusside (SNP) in thoracic aortas **(A, B)** and mesenteric resistance arteries (MRAs) **(C, D)** of C57Bl/6 mice injected with tunicamycin and systemically infused with miR-204 I. Control mice were treated with Veh and infused with miR SC. There were no significant differences between groups. **(E)** Immunoblot quantification for ER stress markers (BIP, P-PERK, and ATF6) in thoracic aortas of mice in **A-D** above. Values are normalized to β-actin and expressed relative to miR SC + Veh.*p<0.05 vs miR SC + Veh, miR-204 I + Tunica, and miR-204 I + Veh. n = 3.

Supplementary Fig. 4. Sirt1 suppresses miR-204-mediated ER stress. Quantification of immunoblots (**A**) and immunofluorescence images with quantification (**D, E**) for ER stress markers (BIP, P-PERK, P-elf2α, CHOP, and ATF6) and Sirt1 in HUVECs transfected with siRNA SIRT1 and treated with tunicamycin. Control cells were transfected with a scrambled siRNA (siRNA SC) and treated with Veh. Images are representative of three independent experiments. Values are normalized to β-actin (for immunoblots) and expressed relative to siRNA SC + Veh. *p<0.05 vs siRNA SC + Veh. n = 3 for immunoblots and n = 10-17 cells for immunofluorescence. Quantification of immunoblots **(B, C**) and immunofluorescence images with quantification (**F, G**) for ER stress markers (BIP, P-PERK, P-elf2α, CHOP, and ATF6) and Sirt1 in HUVECs infected with Sirt1 adenovirus (Ad Sirt1) and treated with Tunica or transfected with miR-204 mimic. Control cells were infected with Ad LacZ and treated with Veh or transfected with miR SC. Values are normalized to β-actin (for immunoblots) and expressed relative to Ad LacZ + Veh (in **B, F, G**) or Ad LacZ + miR SC (in **C**). * p<0.05 vs Ad LacZ + Veh and Ad Sirt1 + Tunica (in **B, F, G**), or Ad LacZ + miR SC and Ad Sirt1 + miR-204 mimic (in **C**). # p<0.05 vs Ad LacZ + Tunica (in **B, F**), or Ad LacZ + miR-204 mimic (in **C**). n = 3 and n = 12-18 cells for immunofluorescence. Quantification of miR-204 **(H)** and TRPM3 **(I)** by qPCR in HUVECs infected with Ad Sirt1 and treated with Tunica. Control cells were infected with Ad LacZ and treated with Veh. MiR-204 is normalized to RNU6 and expressed relative to Ad LacZ + Veh. * p<0.05 vs Ad LacZ + Veh, and Ad Sirt1 + Tunica. n = 3. Immunofluorescence images with quantification for ER stress markers (BIP and CHOP) **(J-K**) and quantification of miR-204 by qPCR (**L**) in HUVECs infected with Ad Sirt1 and transfected with miR-204 mimic. Control cells were infected with Ad LacZ and transfected with miR SC. MiR-204 was normalized to RNU6 and values are expressed relative to Ad LacZ + miR SC. *p<0.05 vs

Ad LacZ + miR SC and Ad Sirt1 + miR-204 mimic. n = 10-21 cells for immunofluorescence. n = 3 for qPCR.

Supplementary Fig. 5. Sirt1 suppresses thapsigargin-induced ER stress. Immunoblots (**A**) and qPCR (**B**) for ER stress markers (BIP, P-PERK, CHOP, ATF4 and ATF6) in HUVECs infected with Ad Sirt1 and treated with thapsigargin (TPS). Control cells were infected with Ad LacZ and treated with Veh. Immunoblots are representative of three independent experiments. Values are normalized to GAPDH and expressed relative to Ad LacZ + Veh. *$p<0.05$ compared to all other groups. n = 3.

Supplementary Fig. 6. Schematic summarizing the roles and interactions of miR-204 and Sirt1 in the regulation of vascular ER stress.

Supp. Fig. 1

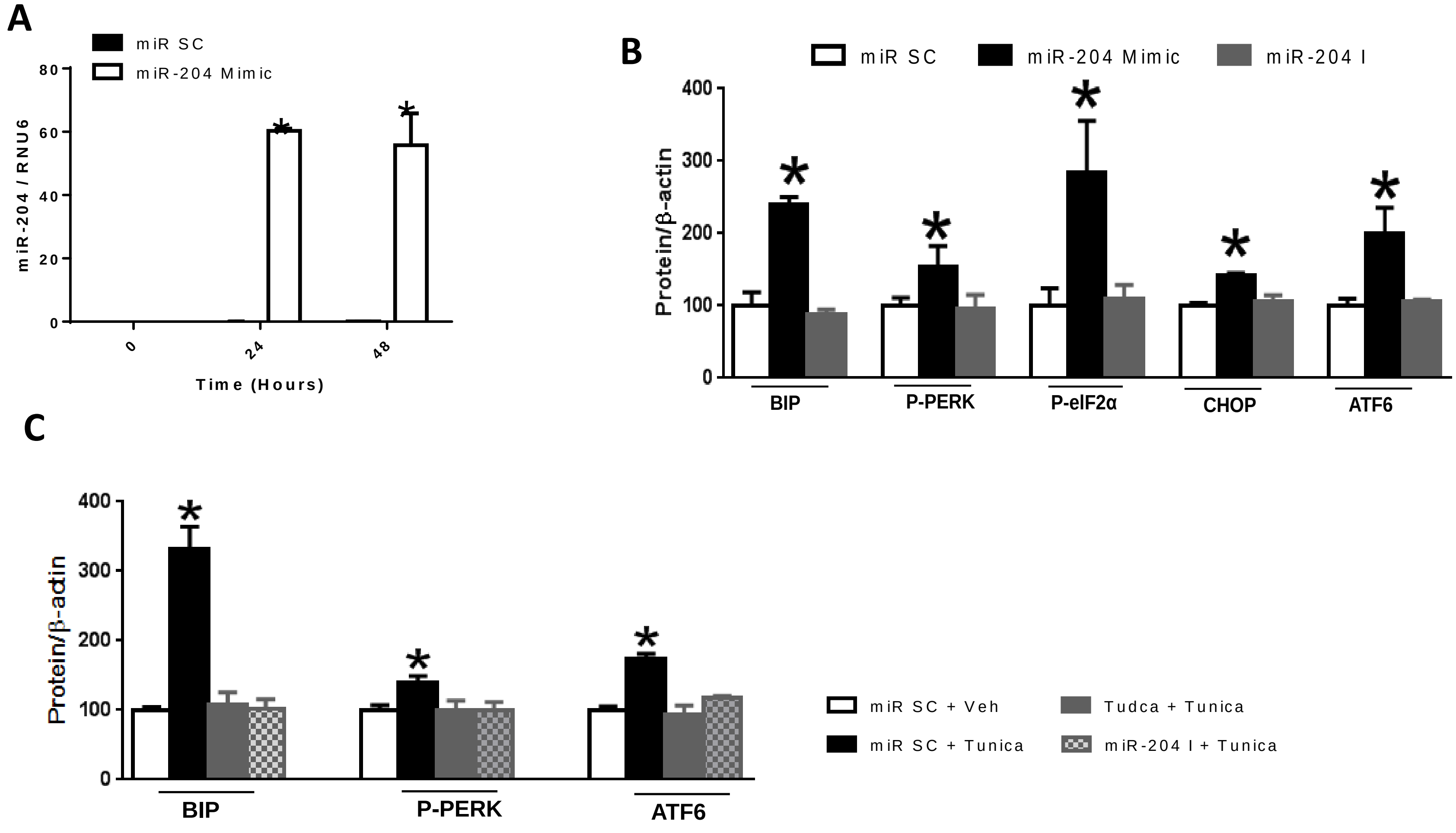
A
miR SC
miR-204 Mimic
miR-204 / RNU6
80
60
40
20
0
24
48
Time (Hours)
*
*
B
miR SC
miR-204 Mimic
miR-204 I
Protein/β-actin
400
300
200
100
0
BIP
P-PERK
P-eIF2α
CHOP
ATF6
C
Protein/β-actin
400
300
200
100
0
BIP
P-PERK
ATF6
miR SC + Veh
miR SC + Tunica
Tudca + Tunica
miR-204 I + Tunica

Supp. Fig. 2

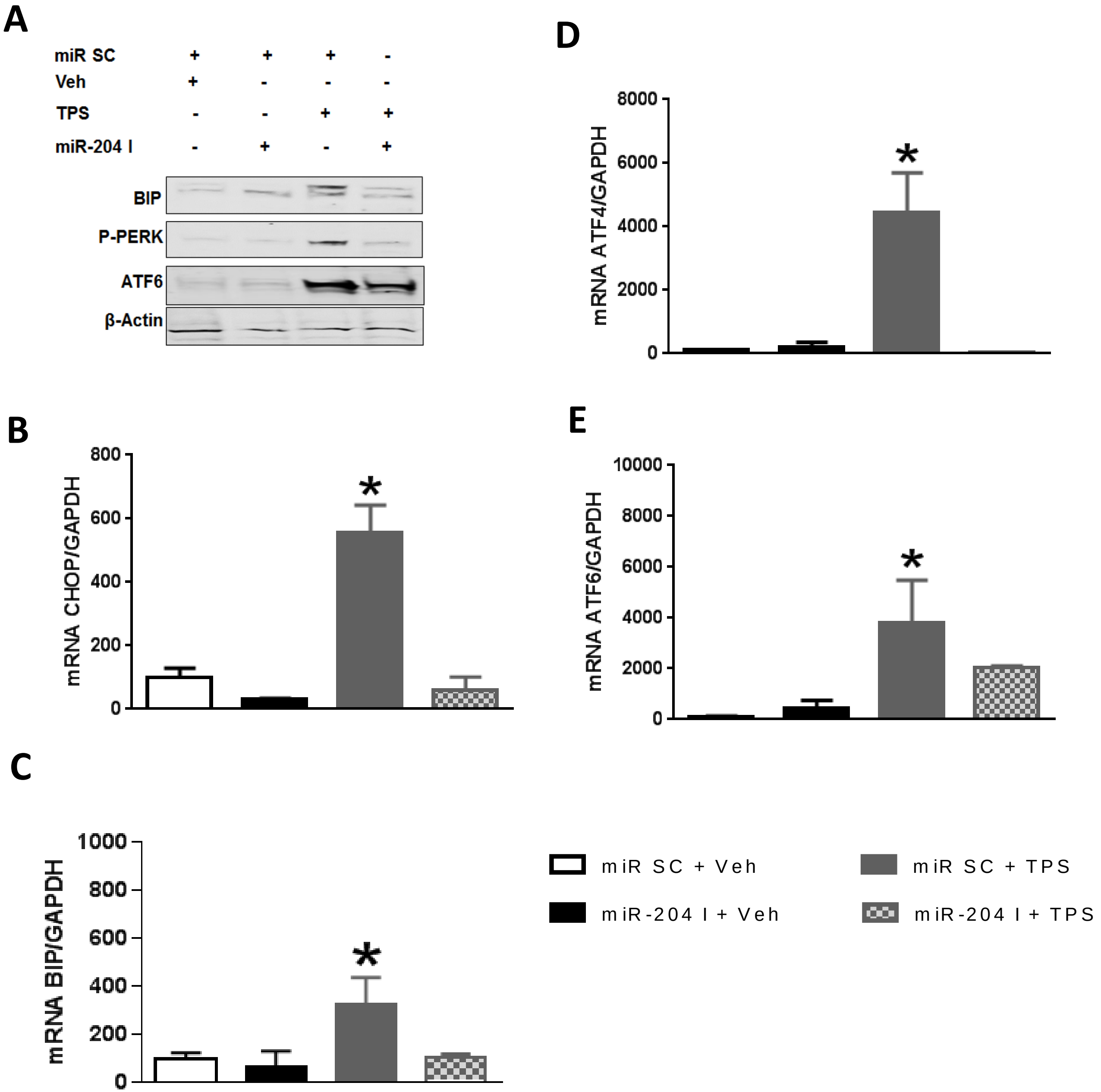

A
miR SC + + + -
Veh + - - -
TPS - - + +
miR-204 I - + - +
BIP
P-PERK
ATF6
β-Actin
B
mRNA CHOP/GAPDH
800
600
400
200
0
*
C
mRNA BIP/GAPDH
1000
800
600
400
200
0
*
D
mRNA ATF4/GAPDH
8000
6000
4000
2000
0
*
E
mRNA ATF6/GAPDH
10000
8000
6000
4000
2000
0
*
miR SC + Veh
miR SC + TPS
miR-204 I + Veh
miR-204 I + TPS

Supp. Fig. 3

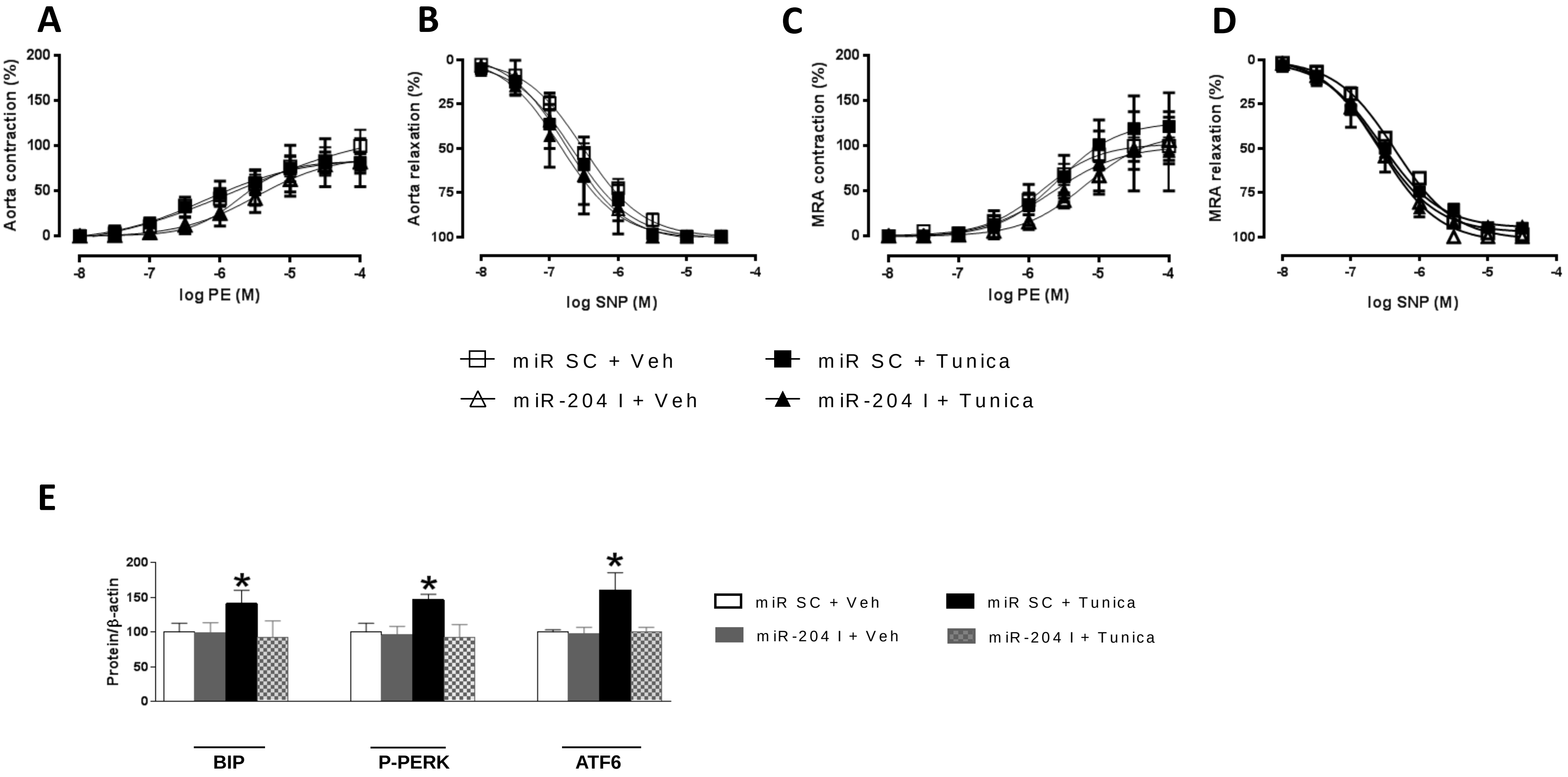
A
B
C
D
Aorta contraction (%)
log PE (M)
Aorta relaxation (%)
log SNP (M)
MRA contraction (%)
log PE (M)
MRA relaxation (%)
log SNP (M)
miR SC + Veh
miR SC + Tunica
miR-204 I + Veh
miR-204 I + Tunica
E
Protein/β-actin
BIP
P-PERK
ATF6
miR SC + Veh
miR SC + Tunica
miR-204 I + Veh
miR-204 I + Tunica

Supp. Fig. 4

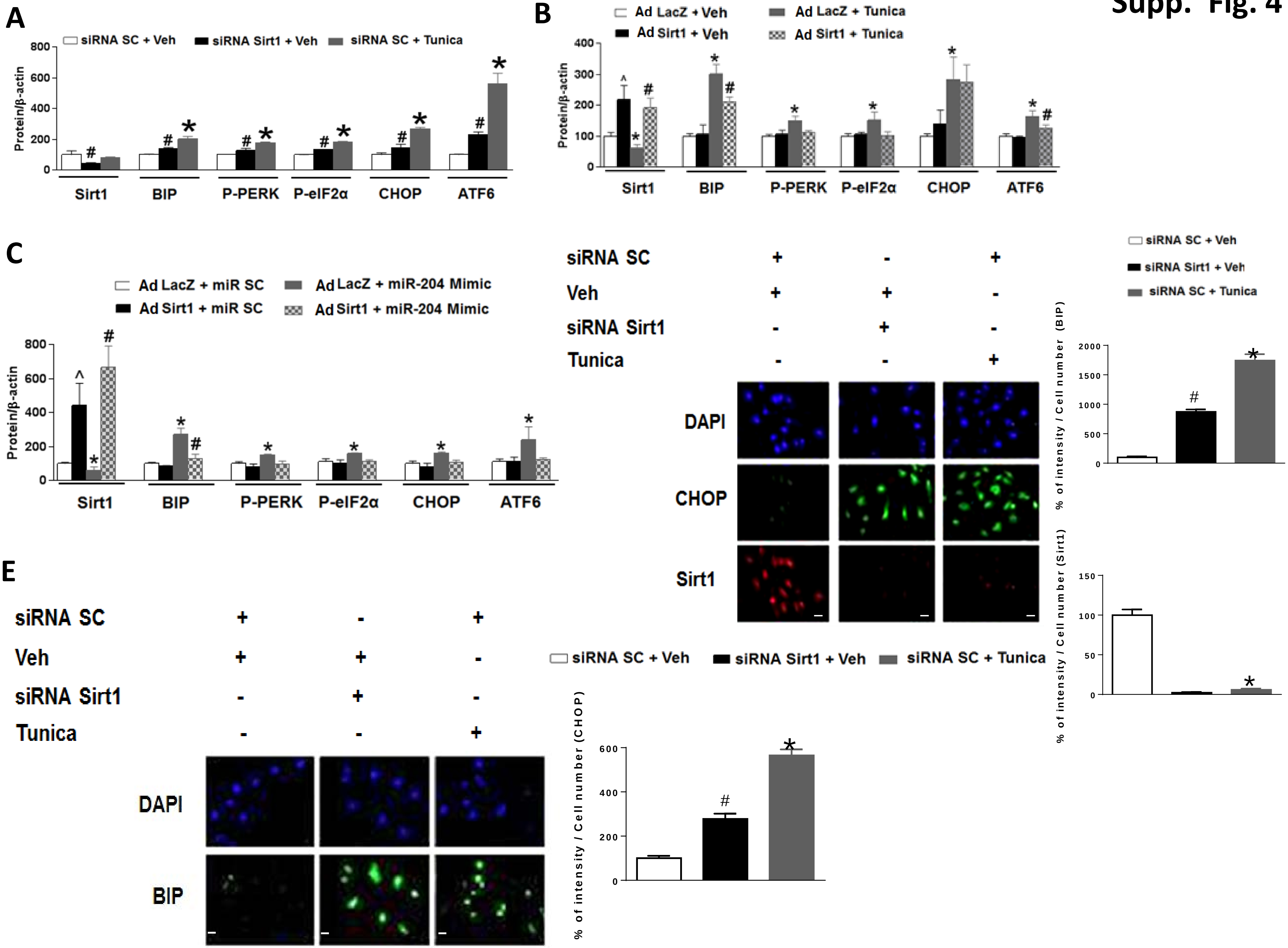
A
siRNA SC + Veh
siRNA Sirt1 + Veh
siRNA SC + Tunica
Protein/β-actin
800
600
400
200
0
Sirt1
BIP
P-PERK
P-eIF2α
CHOP
ATF6
B
Ad LacZ + Veh
Ad Sirt1 + Veh
Ad LacZ + Tunica
Ad Sirt1 + Tunica
Protein/β-actin
400
300
200
100
0
Sirt1
BIP
P-PERK
P-eIF2α
CHOP
ATF6
C
Ad LacZ + miR SC
Ad Sirt1 + miR SC
Ad LacZ + miR-204 Mimic
Ad Sirt1 + miR-204 Mimic
Protein/β-actin
800
600
400
200
0
Sirt1
BIP
P-PERK
P-eIF2α
CHOP
ATF6
siRNA SC + - +
Veh + + -
siRNA Sirt1 - + -
Tunica - - +
DAPI
CHOP
Sirt1
siRNA SC + Veh
siRNA Sirt1 + Veh
siRNA SC + Tunica
% of intensity / Cell number (BIP)
2000
1500
1000
500
0
% of intensity / Cell number (Sirt1)
150
100
50
0
E
siRNA SC + - +
Veh + + -
siRNA Sirt1 - + -
Tunica - - +
DAPI
BIP
siRNA SC + Veh
siRNA Sirt1 + Veh
siRNA SC + Tunica
% of intensity / Cell number (CHOP)
600
400
200
0

Supp. Fig. 4

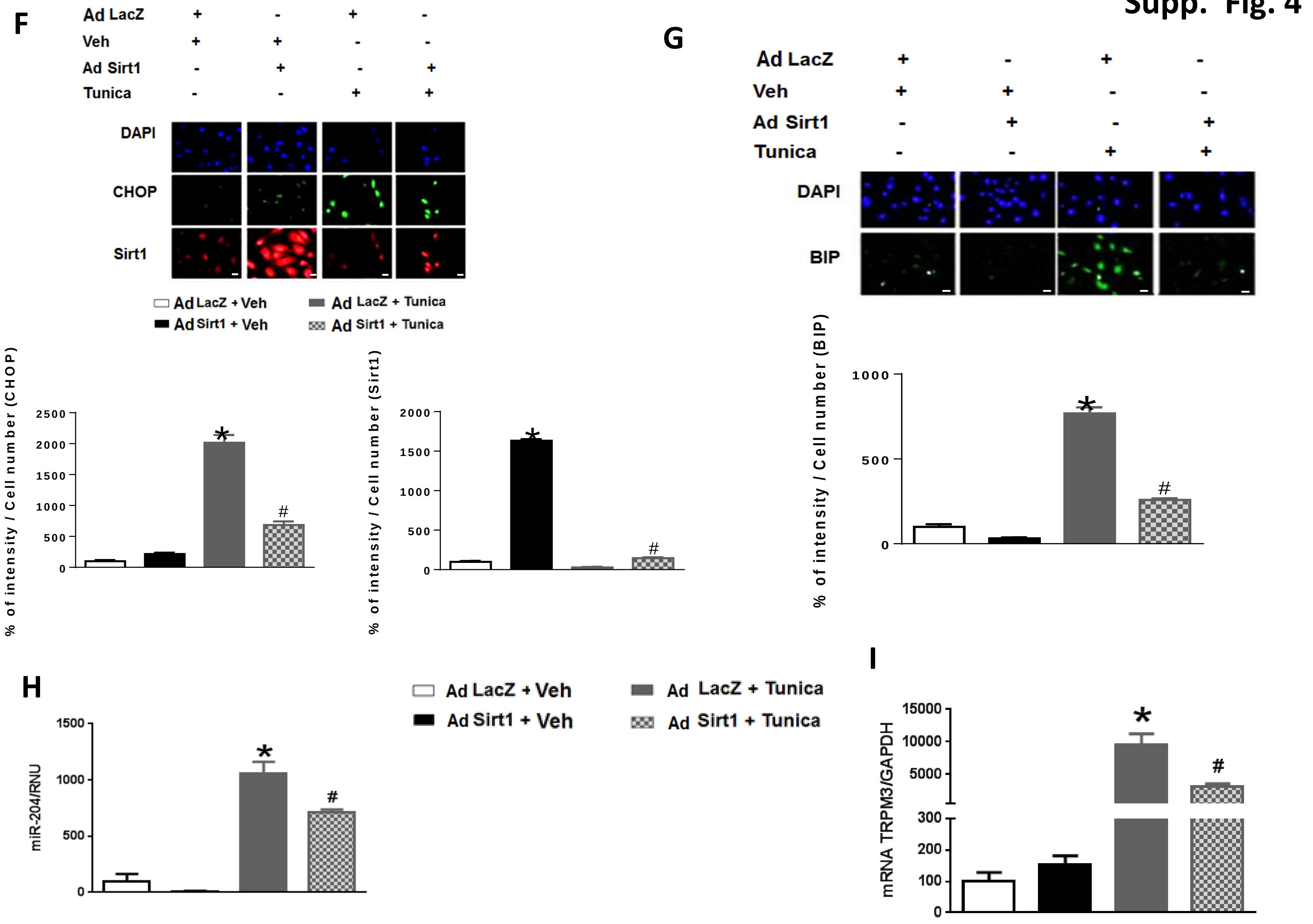
F
Ad LacZ + - + -
Veh + + - -
Ad Sirt1 - + - +
Tunica - - + +
DAPI
CHOP
Sirt1
Ad LacZ + Veh
Ad Sirt1 + Veh
Ad LacZ + Tunica
Ad Sirt1 + Tunica
% of intensity / Cell number (CHOP)
% of intensity / Cell number (Sirt1)
G
Ad LacZ + - + -
Veh + + - -
Ad Sirt1 - + - +
Tunica - - + +
DAPI
BIP
% of intensity / Cell number (BIP)
H
miR-204/RNU
I
mRNA TRPM3/GAPDH

Supp. Fig. 4

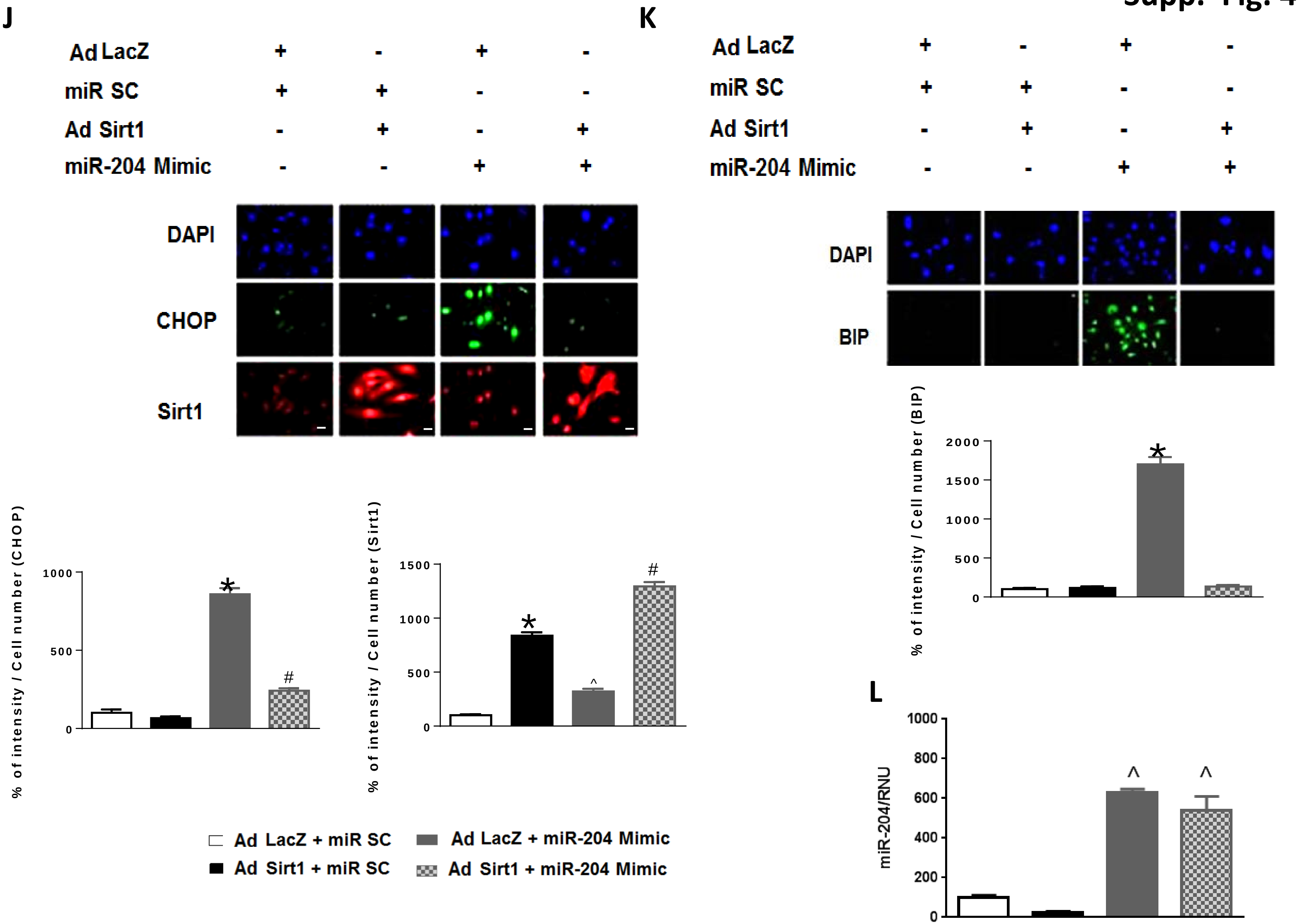
J
Ad LacZ + - + -
miR SC + + - -
Ad Sirt1 - + - +
miR-204 Mimic - - + +
DAPI
CHOP
Sirt1
% of intensity / Cell number (CHOP)
1000
500
0
*
#
% of intensity / Cell number (Sirt1)
1500
1000
500
0
*
^
#
Ad LacZ + miR SC
Ad Sirt1 + miR SC
Ad LacZ + miR-204 Mimic
Ad Sirt1 + miR-204 Mimic
K
Ad LacZ + - + -
miR SC + + - -
Ad Sirt1 - + - +
miR-204 Mimic - - + +
DAPI
BIP
% of intensity / Cell number (BIP)
2000
1500
1000
500
0
*
L
miR-204/RNU
1000
800
600
400
200
0
^
^

Supp. Fig. 5

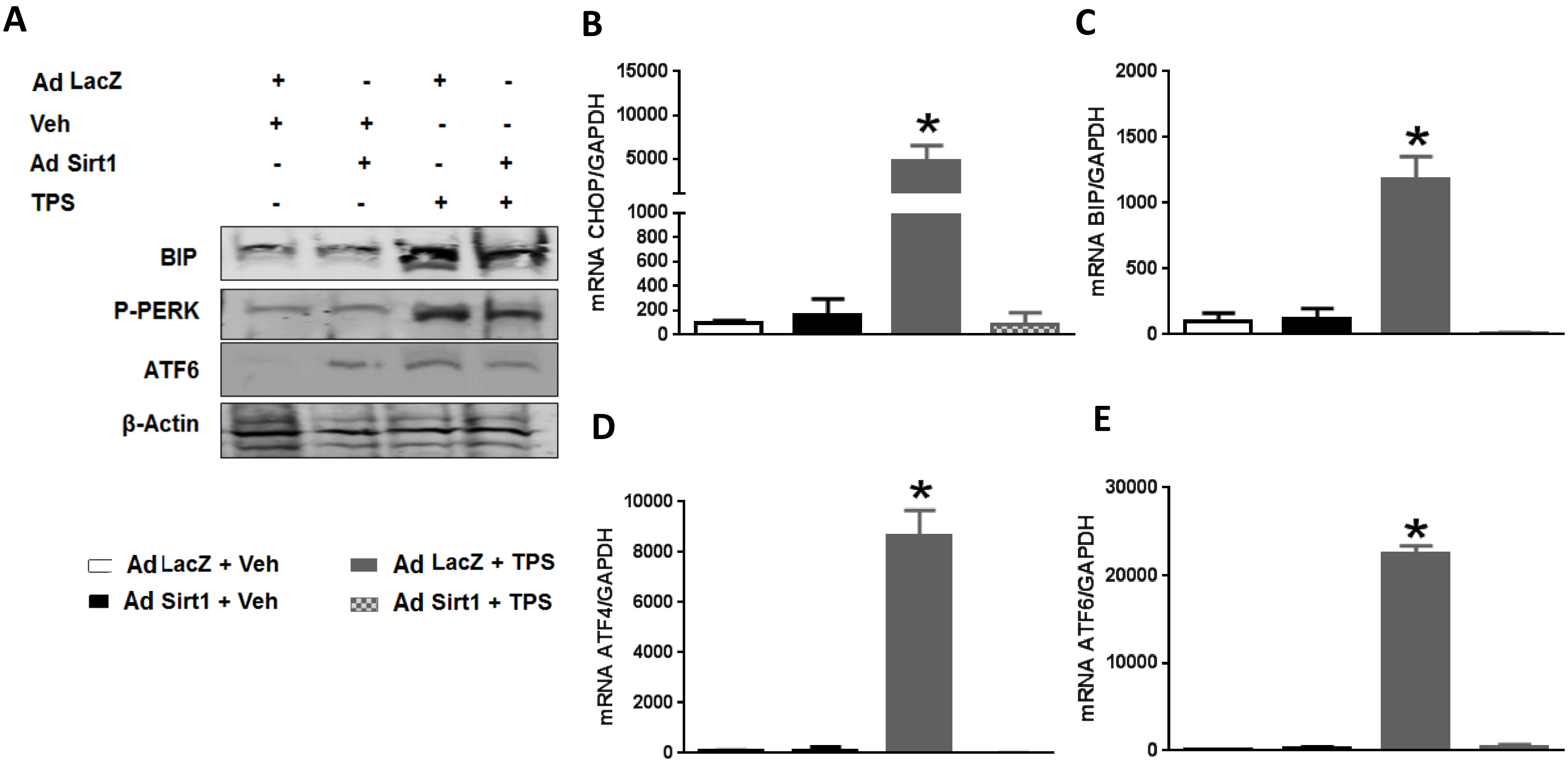
A
Ad LacZ + - + -
Veh + + - -
Ad Sirt1 - + - +
TPS - - + +
BIP
P-PERK
ATF6
β-Actin
Ad LacZ + Veh
Ad Sirt1 + Veh
Ad LacZ + TPS
Ad Sirt1 + TPS
B
mRNA CHOP/GAPDH
15000
10000
5000
1000
800
600
400
200
0
*
C
mRNA BIP/GAPDH
2000
1500
1000
500
0
*
D
mRNA ATF4/GAPDH
10000
8000
6000
4000
2000
0
*
E
mRNA ATF6/GAPDH
30000
20000
10000
0
*

Supp. Fig. 6

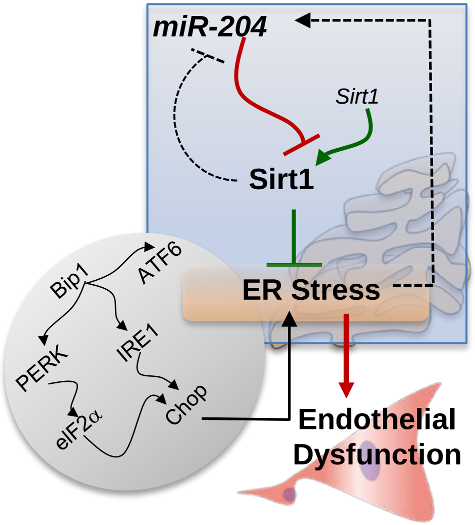

miR-204
Sirt1
Sirt1
ER Stress
Bip1
ATF6
PERK
IRE1
eIF2α
Chop
Endothelial
Dysfunction
